# Selective-Area Epitaxy of InGaAsP Buffer Multilayer for In-Plane InAs Nanowire Integration

**DOI:** 10.3390/ma15072543

**Published:** 2022-03-30

**Authors:** Valentina Zannier, Ang Li, Francesca Rossi, Sachin Yadav, Karl Petersson, Lucia Sorba

**Affiliations:** 1 NEST, Istituto Nanoscienze-CNR and Scuola Normale Superiore, Piazza San Silvestro 12, I-56127 Pisa, Italy; lucia.sorba@nano.cnr.it; 2 Beijing Key Laboratory of Microstructure and Properties of Solids, Beijing University of Technology, Beijing 100124, China; ang.li@bjut.edu.cn; 3 IMEM-CNR, Parco Area delle Scienze 37/A, I-43124 Parma, Italy; francesca.rossi@imem.cnr.it; 4 Center for Quantum Devices and Microsoft Quantum Lab-Copenhagen, Niels Bohr Institute, University of Copenhagen, 2100 Copenhagen, Denmark; sachin.yadav@nbi.ku.dk (S.Y.); karl.petersson@microsoft.com (K.P.)

**Keywords:** selective-area epitaxy, in-plane nanowires, InAs

## Abstract

In order to use III–V compound semiconductors as active channel materials in advanced electronic and quantum devices, it is important to achieve a good epitaxial growth on silicon substrates. As a first step toward this, we report on the selective-area growth of GaP/InGaP/InP/InAsP buffer layer nanotemplates on GaP substrates which are closely lattice-matched to silicon, suitable for the integration of in-plane InAs nanowires. Scanning electron microscopy reveals a perfect surface selectivity and uniform layer growth inside 150 and 200 nm large SiO_2_ mask openings. Compositional and structural characterization of the optimized structure performed by transmission electron microscopy shows the evolution of the major facet planes and allows a strain distribution analysis. Chemically uniform layers with well-defined heterointerfaces are obtained, and the topmost InAs layer is free from any dislocation. Our study demonstrates that a growth sequence of thin layers with progressively increasing lattice parameters is effective to efficiently relax the strain and eventually obtain high quality in-plane InAs nanowires on large lattice-mismatched substrates.

## 1. Introduction

Indium arsenide (InAs) is an ideal material for the realization of high-electron-mobility transistors and low-power-consumption devices, thanks to its low electron effective mass and its excellent transport properties. Moreover, one-dimensional (1D) InAs channels can be proximitized by superconductors to realize topological superconductor networks and quantum devices [1,2,3,4,5]. However, the monolithic integration of InAs in the silicon technology requires the development of a buffer layer accommodating the large lattice mismatch (11.6% between InAs and Si) and to solve the problem of anti-phase domain (APD) formation [6]. The latter can be overcome with the epitaxial growth of a GaP interfacial layer on Si (100) substrates. GaP has the smallest lattice mismatch among all III–V semiconductors with Si (<0.4%), and it has long been recognized as the natural candidate for III–V integration on silicon. Indeed, the epitaxial growth of GaP on Si was demonstrated first in the 1980s [7,8], and much progress has been made in the last years toward the growth of GaP layers free from dislocations, stacking faults, and APD on Si substrates [9,10,11]. These GaP/Si substrates are the ideal templates for the realization of active III–V devices and are now commercially available, even if the mismatch problem remains, especially for InAs and antimony-based compounds. The lattice mismatch can be accommodated through the growth of an engineered buffer layer sequence with varying composition and lattice parameter [10,12,13]. Moreover, nanometer-sized interfaces are known to facilitate the growth of heterostructures with a low dislocation density, exploiting the small interfacial area to allow an efficient elastic strain relaxation. This is a well-known mechanism in vertical nanowires (NWs), where defect-free axial heterostructures can be realized, also combining large lattice mismatched materials [14,15,16]. Indeed, direct integration of vertical III–V NWs on silicon has been demonstrated, and the dislocation density at the interface is strongly reduced as the NW diameter narrows [17]. A similar effect has been observed also for in-plane GaSb NWs grown on GaAs (100) substrates, where the density of threading dislocations at the heterointerface is strongly reduced compared to a GaSb 2D layer grown on the same substrate [12]. For this reason, selective area growth (SAG) is a promising approach for the III–V integration on silicon. However, the direct SAG of materials with large lattice mismatch still resulted in the formation of misfit dislocations [2,13,18,19]. Only for extremely small (≤30 nm) interfaces [20], or by using nano-templates [21,22], could a defect-free growth be obtained. For larger interfaces, only the growth of a buffer layer allowed the improvement of the quality of the topmost layer [13,23].

Here, we combine the SAG approach with a buffer multilayer sequence with increasing lattice parameter to accommodate the strain for the growth of InAs on GaP substrates. In particular, we investigated the growth of GaP/InGaP/InP/InAsP/InAs on SiO_2_-coated GaP substrates by selective-area chemical beam epitaxy (CBE) inside narrow mask openings. We demonstrate that this approach can be adopted for the integration of in-plane InAs NWs on GaP substrates, a potential structure for realizing novel quantum devices. Moreover, the successful SAG on GaP (001) substrates paves the way toward the direct integration of such nanostructures on the silicon platform.

## 2. Materials and Methods

GaP (001) substrates have been used for the SA growth. A 10 nm thick Al_2_O_3_ etch-stop layer was deposited on the substrate by atomic layer deposition (ALD), followed by a 100 nm thick SiO_2_ layer growth by plasma enhanced chemical vapor deposition (PECVD). Electron beam lithography (EBL) was used to define patterns in the resist deposited on the oxide layer. The EBL pattern consists of line openings (trenches) oriented along the [110] substrate direction. Whole-wafer-long lines of different width were realized: nominally 50, 100, 150, and 200 nm. By means of reactive ion etching (RIE), the SiO_2_ layer was totally removed inside the trenches, and the remaining Al_2_O_3_ layer was wet etched with tetramethylammonium hydroxide (TMAH) solution (MF319 developer) just before introducing the substrate into the CBE system. The epitaxial growth was performed in a RIBER Compact 21 CBE chamber. We used trimethylindium (TMIn), triethylgallium (TEGa), tert-butylphosphine (TBP), and tert-butylarsine (TBAs) as metal-organic (MO) precursors, and an optical pyrometer was used to measure the sample temperature with an accuracy of ±10 °C. We first performed a thermal annealing, keeping the sample for 20 min at 580 °C under TBP flux (line pressure 1 Torr) in order to deoxidize the GaP substrate in the patterns. Then the temperature was lowered to 550 °C, and the growth was started. First, we grew a GaP layer using TEGa and TBP line pressures of 0.3 and 1 Torr, respectively. Then we grew the InGaP/InP/InAsP/InAs layer sequence, exploring different growth temperatures and MO line pressures, as will be described in the following. Scanning electron microscopy (SEM) analysis was performed after each growth step in a Zeiss Merlin field emission microscope operated at 5 KeV by acquiring top- and tilted-view (90°) images. For the 90°-tilted images, used also to measure the thicknesses of the layer grown into the patterns, the samples were cleaved and analyzed in cross-section. Crystal structure, elemental composition, interface quality, and strain mapping of the optimized complete structure were studied by scanning transmission electron microscopy (STEM) with a FEI-Titan-Themis microscope operated at 300 keV, equipped with probe aberration corrector, and a set of energy-dispersive X-ray spectroscopy (EDX) detectors. The camera length was set to 195 mm, and the collection angle was set to 40–200 mrad. Cross-sectional lamellae of the selected structures were cut by focused ion beam (FEI-Helios 650) with a Pt deposited protection layer.

## 3. Results and Discussion

**Figure 1:**
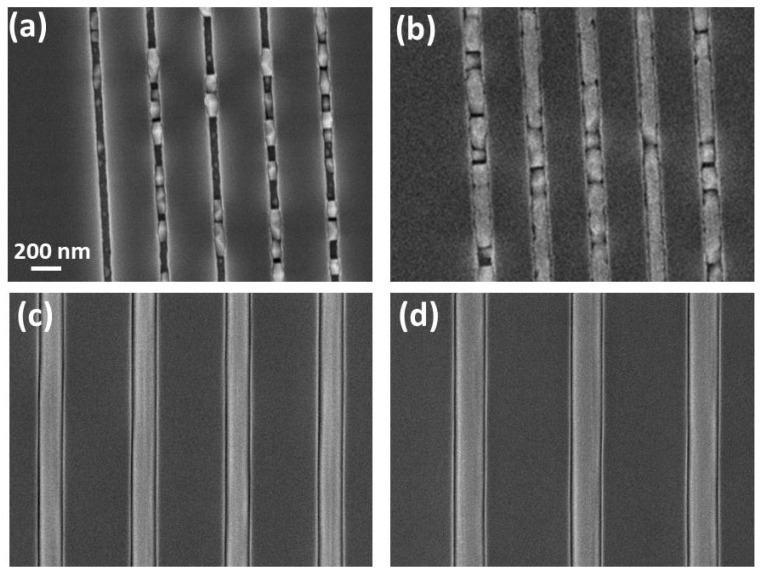
Top-view SEM images of the sample after 60 min GaP growth at 550 °C substrate temperature in the different patterns with nominal mask openings of 50 nm (**a**), 100 nm (**b**), 150 nm (**c**), and 200 nm (**d**). The scale bar (200 nm) is the same for all panels.

First, the growth of GaP buffer layers on (001) GaP substrates into trench patterns of different width, from 50 to 200 nm, was tested. Figure 1 shows the top-view SEM images of the different patterns after 1 h of GaP growth. It is quite clear that the GaP growth occurs only into the trenches, while there is no deposition on the SiO_2_ mask that looks perfectly clean. This confirms that CBE is a highly suitable growth technique for the SAG of III–V semiconductors on dielectric-patterned substrates [24]. However, we found that the growth in the thinner trenches (with a nominal width of 50 and 100 nm: panels (a) and (b), respectively) resulted in separated GaP islands, while the growth into the wider trenches (with nominal width of 150 and 200 nm: panels (c) and (d), respectively) resulted in a continuous GaP layer filling the whole trenches for their total length. The different morphology can be either a result of different growth modes for mask openings of different width or a possible consequence of a less efficient etching procedure for very thin lines. Since a detailed investigation and optimization of the process for the thin trenches was beyond the scope of the present paper, in the following we focus our attention only on the larger trenches that are still suitable for the growth of high-quality thin InAs layers, as will be shown. Here, we could measure a GaP thickness of 120 nm, resulting in a growth rate of 2 nm/min.

**Figure 2:**
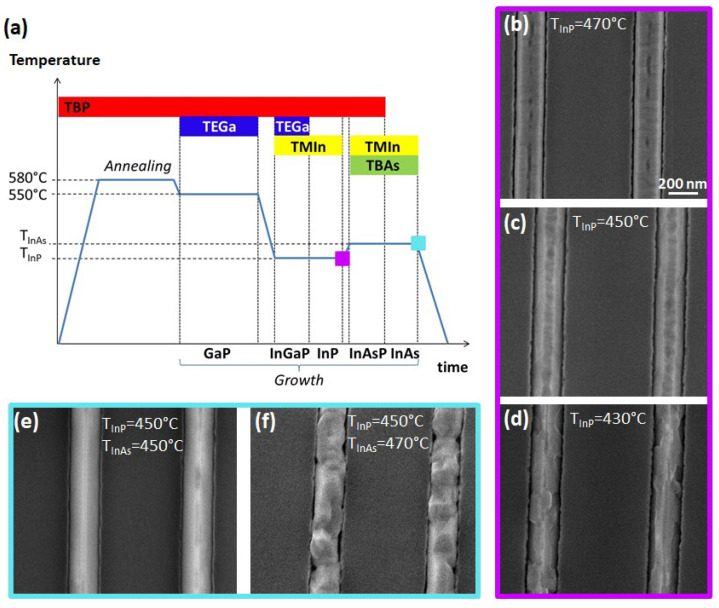
Growth protocol and samples obtained at different stages of the growth sequence: at the end of InP layer growth (violet framed) and at the end of InAs growth (cyan framed) for different temperatures. (**a**) Schematic view of the complete growth process. (**b**–**d**) Top-view SEM images of the GaP/InGaP/InP layers grown on the 200 nm wide trenches for different T_InP_: 470 °C (**b**), 450 °C (**c**), and 430 °C (**d**). (**e**,**f**) Top-view SEM images of the GaP/InGaP/InP/InAsP/InAs samples obtained at T_InP_ = 450 °C and T_InAs_ = 450 °C in (**e**) and 470 °C in (**f**). The scale bar (200 nm) is the same for all panels.

In the next steps, we investigated the growth of the InGaP/InP/InAsP/InAs multilayer sequence on the GaP buffer. It is known that the growth temperature window for InP, InGaP, and InAsP alloys [25,26] is lower than the GaP growth temperature [27]. Therefore, we developed a growth protocol as depicted in Figure 2a: we first grew 35 min of GaP at 550 °C, resulting in 70 nm thick layer, and then we lowered the substrate temperature under P flux. Once reached the growth temperature T_InP_, we grew in sequence the InGaP layer and the InP layer at constant temperature (T_InP_) and without any growth interruption. In particular, we grew 10 min of InGaP using TMIn, TEGa, and TBP line pressures of 0.3, 0.3, and 1 Torr, respectively, followed by 10 min of InP with TMIn and TBP line pressures of 0.3 and 1 Torr, respectively. We tested different values of T_InP_, as shown in Figure 2b–d. We found that when the growth temperature is too high (T_InP_ = 470 °C), the InGaP/InP layer is thin (around 50 nm) and not uniform, showing some holes in the (001) top facet (Figure 2b). On the other hand, when the temperature is too low (T_InP_ = 430 °C), the InGaP/InP layer is thicker (around 80 nm as average), but many steps are visible on both the top and the side facets, resulting in a very rough profile (Figure 2d). The growth at T_InP_ = 450 °C results in the best morphology showing quite smooth surfaces, with a total thickness around 140 nm, which means 70 nm of the InGaP/InP layers (Figure 2c). It should be noticed that the (001) flat facet narrows, while the {111} inclined sidewalls widen, in comparison to the GaP layer underneath. The profile evolution during the different material growth will be discussed more in detail in the next session. 

Finally, we grew the InAsP and InAs layers on top of the optimized GaP/InGaP/InP sequence obtained at T_InP_ = 450 °C. We grew the two materials at constant temperature (T_InAs_) and without any growth interruption. We tried two different values of T_InAs_: same as the InGaP/InP growth temperature (T_InAs_ = T_InP_ = 450 °C) and higher (T_InAs_ = T_InP_ + 20 °C = 470 °C). In the first case, we directly switched from InP to InAsP growth, while in the second one we increased the temperature under P flux before starting the InAsP layer growth. Once reached T_InAs_, we grew the InAsP layer for 10 min with TMIn, TBAs, and TBP line pressures of 0.3, 0.3, and 1.2 Torr, followed by 10 min of InAs at the same temperature, using TMIn and TBAs line pressures of 0.3 and 1 Torr, respectively. Panels (e) and (f) of Figure 2 show the top-view SEM images of the samples obtained at the different T_InAs_. 

We found that the InAsP/InAs growth at higher temperature (T_InAs_ = 470 °C) results in a very rough layer with many steps and holes. Conversely, the growth at T_InAs_ = 450 °C results in a uniformly thick layer with smooth sidewalls. The (001) top facet shrinks and tends to disappear at the end of the InAs topmost layer growth, while the two inclined {111} facets widen, so that the final in-plane nanowire structure has a quasi-triangular cross-sectional profile.

**Figure 3:**
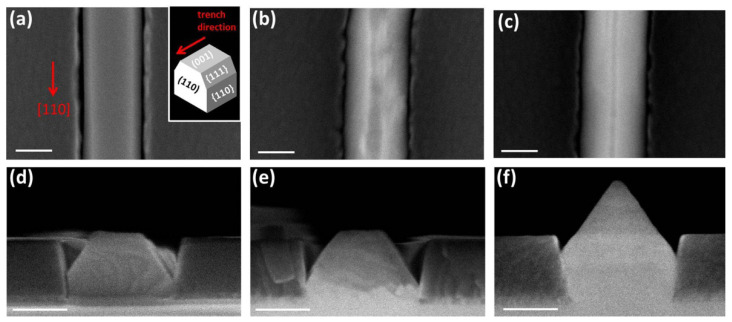
Top-view (**a**–**c**) and 90° tilted-view (**d**–**f)** of the structures grown inside 200 nm wide trenches after different growth sequences: 60 min of GaP at 550 °C (**a**,**d**), 35 min GaP at 550 °C followed by 10 min InGaP and 10 min InP at 450 °C (**b**,**e**) and 35 min GaP at 550 °C + 10 min InGaP + 10 min InP + 10 min InAsP + 10 min InAs at T_InAs_ = T_InP_ = 450 °C (**c**,**f**). The scale bar in each panel corresponds to 100 nm. The inset in panel (**a**) represents a schematic 3D model of a nanowire with trapezoidal cross-section grown inside the [110]-oriented trenches with the different facets indicated.

The evolution of the facets is more clear in Figure 3, where we show the top-view (a–c) and the 90° tilted-view (i.e., the cross sections) (d–f) of the structures after each growth step: GaP (a,d), GaP/InGaP/InP obtained at T_InP_ = 450 °C (b,e), and GaP/InGaP/InP/InAsP/InAs grown at T_InAs_ = T_InP_ = 450 °C (c,f).

The GaP layer has a trapezoidal cross-section with two small vertical {110} facets, a wide (001) top facet, and two inclined {111} facets on the top edges. This morphology has been reported also for in-plane GaSb [19] and GaAsSb [13] nanowires grown into 100–300 nm wide trenches on GaAs (001) substrates, and it has been attributed to a combination of surface energy minimization, constraints due to the mask confinement, and kinetically driven effects. After the growth of the InGaP/InP layers on top of GaP, the (001) top facet narrows, suggesting that the growth rate of the {111} facets is higher. Finally, with the following InAsP and InAs layers growth, the (001) top facet tends to disappear and the growth front has a tapered profile, consisting almost only of the two inclined {111} facets. Some structures still have a portion of the (001) facet or few additional inclined facets, but the two {111} are always the largest developed facets in the final structures. This is consistent with the calculated surface energies for InAs crystallites grown under As-rich conditions that show the {111} family plane as the lowest energy surface [20,28]. Moreover, it has been demonstrated for InAs islands grown on GaAs (001) substrates that a pyramidal shape is almost fully relaxed at the top, compared with a truncated island with a flat top facet [28]. These considerations can explain the cross-sectional profile of our final structure, which is determined by the minimum of the total energy, given by the sum of the elastic energy and the surface energies.

**Figure 4:**
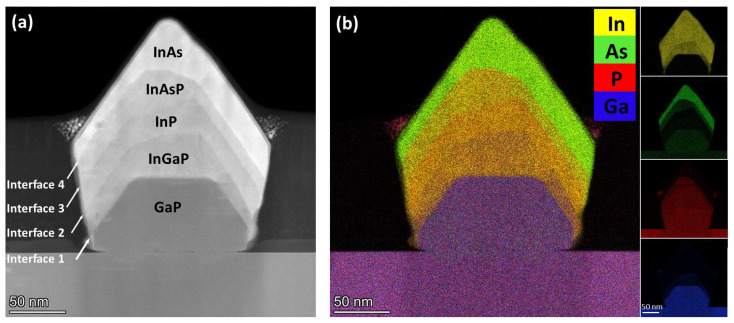
(**a**) STEM-HAADF image of the entire heterostructure in cross-section. The different interfaces are indicated by white arrows and numbered from 1 to 4, following the growth sequence. (**b**) Overlapped EDX map of the same heterostructure depicted in (**a**) with the single element maps as insets on the right.

The final GaP/InGaP/InP/InAsP/InAs optimized heterostructure has been deeply analyzed in order to characterize the chemical composition of each layer, the crystal structure, the quality of the heterointerfaces, and the strain field across the heterointerfaces. In particular, FIB cuts perpendicular to the trenches were realized and the cross-sectional lamellae analyzed by STEM in [110] zone axis. Figure 4 shows a representative structure. Panel (a) is the STEM-HAADF image, where the different Z contrast makes it possible to recognize the different materials. It is clear that the (001) flat top facet behaves as a stable growth front only for the GaP growth. Indeed, the following InGaP layer already shows the inclined facets growing faster, probably with a slightly different growth rate, while the flat top facet narrows and shows some roughening and steps. This morphology propagates also in the InP and InAsP layers, while the growth of the InAs top layer suppresses the (001) flat facet and the growth front propagates almost only with the two inclined {111} facets, which become equivalent in width. It is worth noticing that, at the end of the InAs growth, the structure has a symmetric profile and quite smooth side facets, despite the residual roughness present after the InGaP/InP/InAsP growth sequence. The InAs layer thickness ranges from 10 to 30 nm in the different directions. The chemical composition of each layer was evaluated by the quantitative EDX analysis. Panel (b) of Figure 4 shows the overlapped EDX elemental maps (P, Ga, As, In) of the same structure reported in (a), and the single element maps are reported in the insets. From the analysis of the EDX data (see Supplementary Material S1 for the details), we found that the structure consists of GaP/In_0.65_Ga_0.35_P/InP/InAs_0.45_P_0.55_/InAs and that the chemical composition is uniform within each single layer.

**Figure 5:**
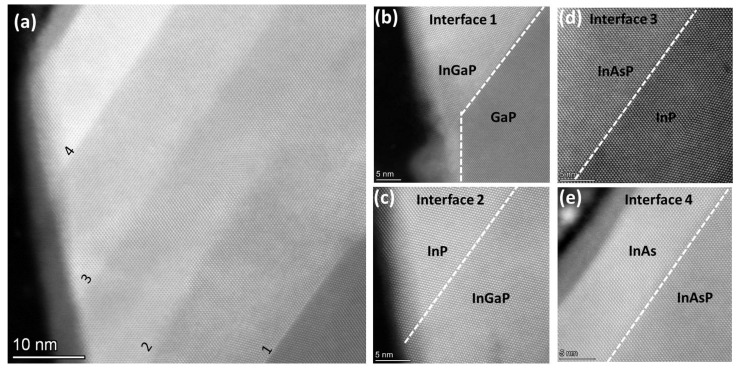
(**a**) HRSTEM image of a portion of the structure containing all of the heterointerfaces as indicated (1–4) on the (111) inclined plane. (**b**–**e**) HRSTEM images of a portion of each heterointerface.

We analyzed the multilayer structures by high-resolution STEM-HAADF (HRSTEM), as shown in Figure 5 and also in Section S2 of the Supplementary Material. This inspection highlights that the epitaxial relation between each material is well preserved and the whole structure has a high crystalline quality, despite the different facets developed and the thickness of each layer, which is often non-uniform in the different crystallographic directions. 

The layer sequence is clearly identifiable by a sharp Z contrast variation, confirming the controlled growth process and the abrupt chemical variation at the heterointerfaces. This is supported also by the EDX line profiles in different directions (see Supplementary Material S1), which show that the intermixing at the interfaces always occurs within 4 nm. It is worth mentioning that this value could be overestimated due to the comparable spatial resolution of the EDX method and to a drift-induced broadening (the collection time of each whole map is about 45 min).

All of the interfaces are flat in the <111> direction, except the InGaP/InP interface (indicated in Figure 5 as number 2), where some roughening occurs. This could be ascribed to an over-growth of the InGaP layer, with the consequent formation of some steps at the interface. Further, the high-resolution imaging shows a quite frequent occurrence of twinning, due to a 60° rotation around the normal to the {111} twinning plane. In Figure 5, these {111} twins are visible in the GaP (Figure 5a, bottom right), InGaP (Figure 5a bottom right and Figure 5b near the interface), and InP (Figure 5c upper left and Figure 5d bottom right) layers, but the inspection of several structures showed that they randomly affect all of the layers. This finding is consistent with the widely reported observation of twinning as characteristic of III–V semiconductors in the <111> direction [29,30,31]. These stacking faults are related to the growth mechanism, usually indicating an island growth mode. It has been shown that the twin density is related to the size of the nuclei [30] and that growth parameters such as growth temperature and beam flux ratio can be used to control the formation of such defects [29,30].

**Figure 6:**
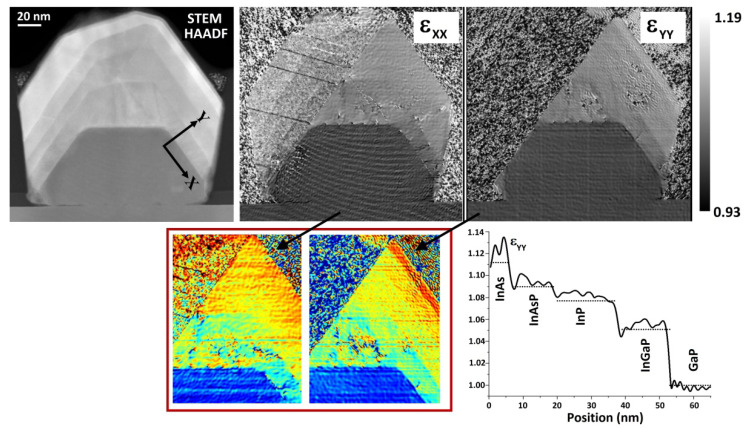
STEM-HAADF image and corresponding ε_xx_, ε_yy_ strain maps. The x and y directions are oriented parallel and perpendicular to the interfaces, respectively, as sketched in the first panel. The bottom line shows a colored and magnified view of the strain maps and the integrated line profile of ε_yy_ taken across the heterointerfaces.

To analyze the strain distribution in the cross-section, STEM-HAADF images were processed by geometric phase analysis (GPA). Figure 6 shows the obtained maps for the strain components parallel (ε_xx_) and perpendicular (ε_yy_) to the heterointerfaces. Only a portion of the heterostructure is visible, due to twinning that causes the crystalline lattice on the left side of the structure to give reciprocal space spots in different positions with respect to its right side. The values start from 1, corresponding to GaP [32]. Looking at the ε_yy_ map, an abrupt change is observed across each interface, allowing one to clearly identify the complete layers sequence. Looking instead at the ε_xx_ map, a sharp contrast is found at the first two interfaces, i.e., GaP/InGaP and InGaP/InP, where a few dislocations occur, while a gradual increase takes place in the topmost material (InP/InAsP/InAs layers). This observation of different behaviors between ε_xx_, increasing by gradual increments, and ε_yy_, increasing by sharp jumps, is indicative of some residual tetragonal distortion in the topmost layers [33]. However, this distortion is indeed small. In fact, the integrated line profile of ε_yy_ (Figure 6 bottom right) shows a good match between the experimental values (solid line) and the expected values (dotted segments), calculated from the ratio to GaP of the relaxed lattice parameters of each layer, assuming a composition profile as measured by EDX spectroscopy. Overall, the strain analysis highlights that the adopted growth approach by buffer layers in the sequence GaP/InGaP/InP/InAsP/InAs is effective to have an efficient strain relaxation.

Clearly, the strain relaxation depends on the buffer layer thickness and on the interface size. We can speculate that thinner trenches would result in even better strain relaxation; therefore, we could expect dislocation-free structures for smaller trench sizes, after optimizing the fabrication process and the growth mode on these narrow mask openings. Concerning the buffer multilayer thickness, we can guess that thinner InGaP/InP/InAsP layers might result in a better accommodation of the elastic strain, possibly resulting in a fully coherent growth.

## 4. Conclusions

We have grown a GaP/InGaP/InP/InAsP multilayer structure on SiO_2_-coated GaP (001) substrate by selective-area chemical beam epitaxy, and we have demonstrated that this nanotemplate is suitable for the epitaxial growth of in-plane InAs nanowires of very good crystal quality. In particular, we could obtain chemically uniform layers with sharp and well-defined heterointerfaces in the <111> directions. Most importantly, the lattice mismatch is relaxed mainly through elastic mechanisms, and the topmost InAs layer is free from any dislocation. We strongly believe that this approach can be adopted for the integration of in-plane InAs NWs on large lattice mismatched substrates and, in particular, on the silicon platform, for the realization of optoelectronic and quantum devices. 

## Data Availability

Data are contained within the article and the Supplementary Material.

## Supplementary Materials

The following supporting information can be downloaded at: https://www.mdpi.com/article/10.3390/ma15072543/s1, S1: EDX analysis and chemical composition, S2: High Resolution TEM analysis.
