# Supplementary material for: Selective-Area Epitaxy of InGaAsP Buffer Multilayer for In-Plane InAs Nanowire Integration
Source: Materials (Basel). 2022 Mar 30;15(7):2543. doi: 10.3390/ma15072543 (PMC8999517; doi:10.3390/ma15072543)
Supplement: Supplementary file 1 [file materials-15-02543-s001.zip › materials-1653243-supplementary.pdf]

Supplementary Materials

Selective-area epitaxy of InGaAsP buffer multilayer for in-plane InAs nanowire integration

Valentina Zannier [1,*], Ang Li [2], Francesca Rossi [3], Sachin Yadav [4], Karl Petersson [4] and Lucia Sorba [1]

[1] NEST, Istituto Nanoscienze-CNR and Scuola Normale Superiore, Piazza San Silvestro 12, I-56127 Pisa, Itlay.
[2] Beijing Key Laboratory of Microstructure and Properties of Solids, Beijing University of Technology, 100124 Beijing, China.
[3] IMEM-CNR, Parco Area delle Scienze 37/A, I-43124 Parma, Italy.
[4] Center for Quantum Devices and Microsoft Quantum Lab-Copenhagen, Niels Bohr Institute, University of Copenhagen, 2100 Copenhagen, Denmark
* Correspondence: valentina.zannier@nano.cnr.it; Tel.: +39050509123

S1. EDX analysis and chemical composition

The chemical composition of each layer has been determined by EDX analysis of the cross-sectional lamellae of the nanostructures. In particular, Ga L, In L, P K, As L lines have been selected for the quantification. From the intensity ratio of the elemental lines we could quantify the chemical composition of each layer and we found that that the structure consists of GaP/$In_{0.65}Ga_{0.35}P$/InP/$InAs_{0.45}P_{0.55}$/InAs. Below we show the EDX map and the elemental line profile of the structure through all the interfaces, in <001> direction (Figure S1.1) and the STEM-HAADF images of magnified portions of the structure in two <111> directions, together with the correspondent EDX line profiles (Figure S1.2):

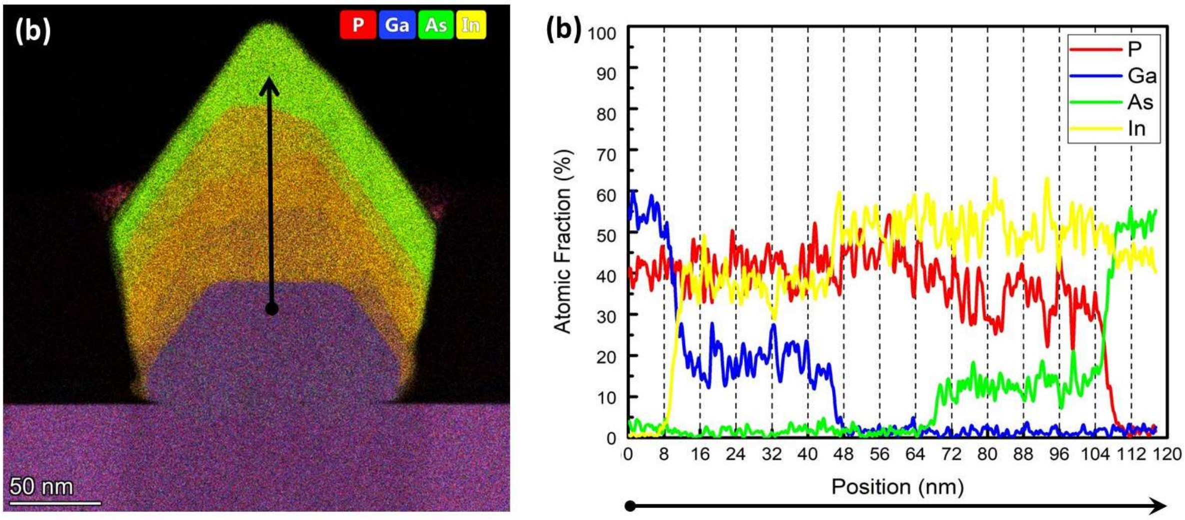

Figure S1.1. (**a**) EDX map of the whole structure and (**b**) elemental line profile in (001) direction across the interfaces as indicated by the black arrow.

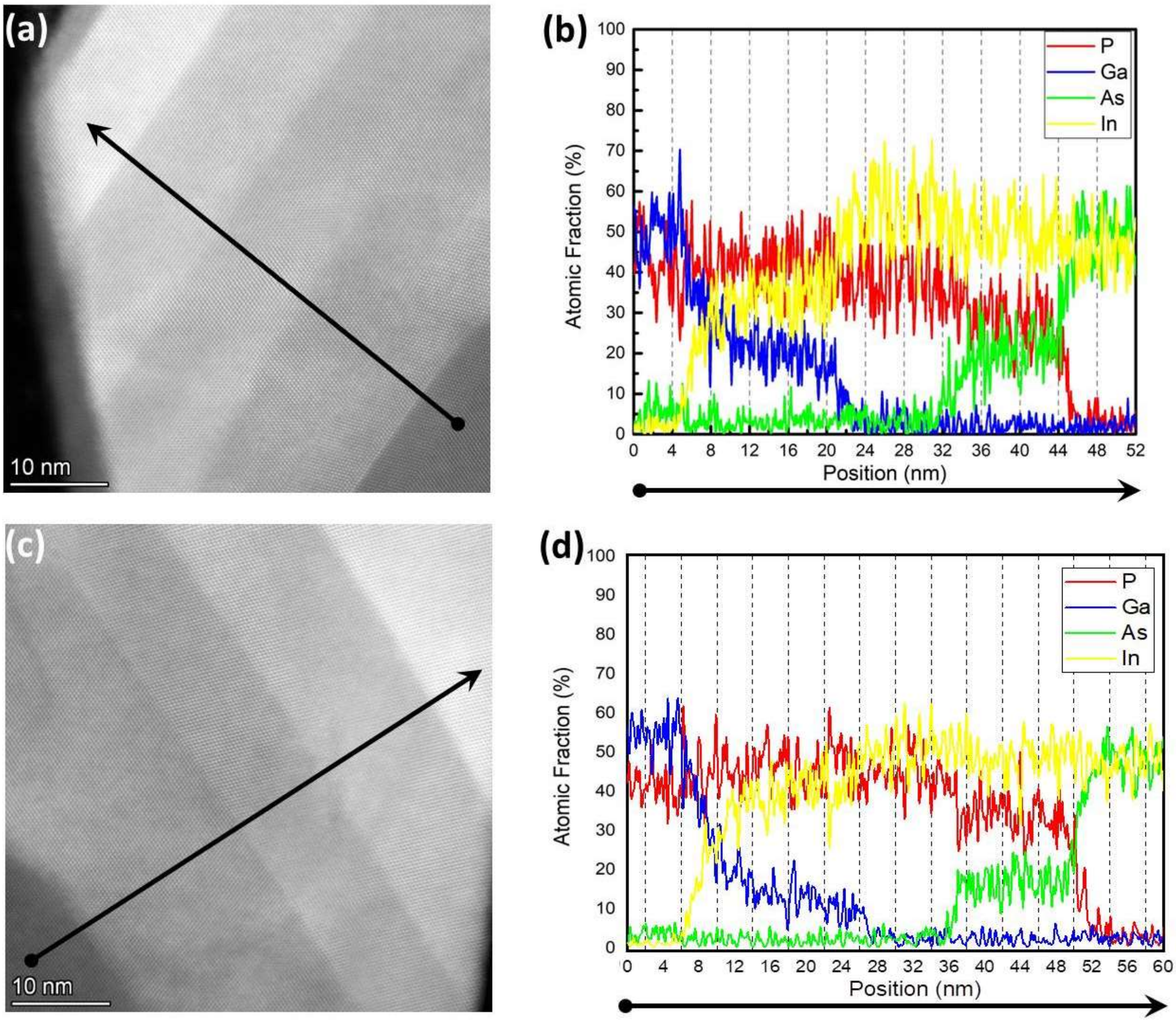

Figure S1.2. (**a,c**) STEM-HAADF images of a portion of the structure containing all the heterointerfaces in two different <111> directions and (**b,d**) corresponding element distribution line profiles acquired be EDX. The profiles start from the GaP core and are measured across the different layers as indicated by the black arrows.

S2. High Resolution TEM analysis

Figure S2.1 shows the STEM-HAADF images of another structure, where the InAs topmost layer exhibits the two {111} facets but also an additional inclined facet, that probably is a result of the non-complete suppression of the (001) growth front. However, as in the structure shown in Figure 5 of the main text, all the interfaces are sharp and abrupt. Some twin planes are also visible in different directions, propagating through the different layers.

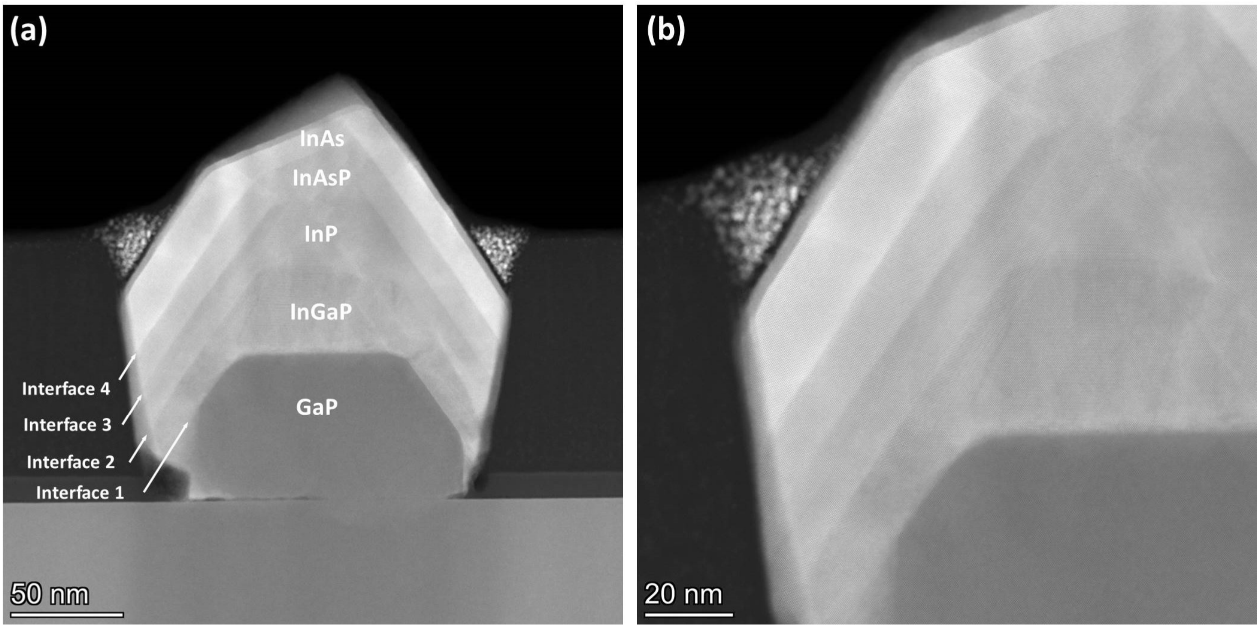

Figure S2.1. Low (**a**) and high (**b**) magnification cross section STEM-HAADF image of another individual heterostructure.

Figure S2.2 shows the HRTEM image of the heterointerfaces in the {111} plane of another structure, highlighting the high crystal quality and the interface sharpness. A twin plane is also visible, as indicated by the orange dotted line.

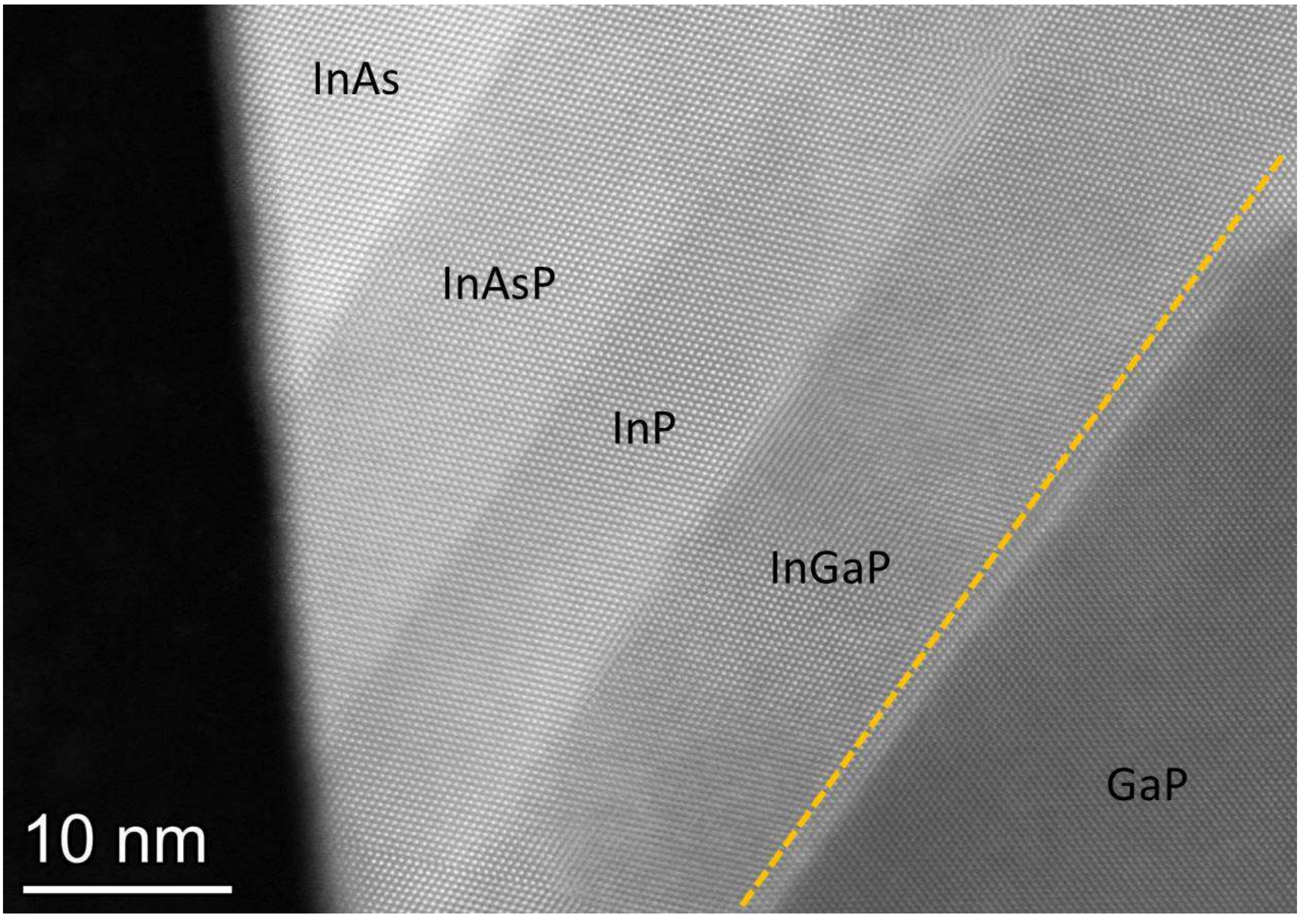

Figure S2.2. Cross section HRSTEM-HAADF image of a portion of another heterostructure with the interfaces in <111> direction well visible. The orange dotted line indicates a twin plane present in the InGaP layer.
